# Mating strategies of *Vitex negundo* L. var. *heterophylla* (Franch.) Rehder (Lamiaceae): A mixed mating system with inbreeding depression

**DOI:** 10.1002/ece3.10927

**Published:** 2024-02-26

**Authors:** Qing Zhang, Jilin Zhang, Xiaohan Sun, Feng Wang, Renqing Wang, Hui Wang, Peiming Zheng

**Affiliations:** ^1^ Institute of Ecology and Biodiversity, School of Life Sciences Shandong University Qingdao China; ^2^ Shandong Provincial Engineering and Technology Research Center for Vegetation Ecology Shandong University Qingdao China; ^3^ Qingdao Forest Ecology Research Station of National Forestry and Grassland Administration Shandong University Qingdao China

**Keywords:** artificial pollination, fruit set, inbreeding depression, mating system, reproductive success, seed germination

## Abstract

Plant reproductive ecology is one of the research hotspots in ecology. With the increasing attention paid to the conservation of plant diversity, the research on reproductive characteristics and flowering biological characteristics of more species has attracted more attention. However, plant reproduction is affected by multiple interacting factors such as pollen limitation and resource availability. *Vitex negundo* var. *heterophylla* (Franch.) Rehder (Lamiaceae) is a significant species for water and soil conservation. Previous studies have revealed its mating system by the biological characteristics of flowering and SSR markers, but its reproductive strategies remain to be further studied. We evaluated reproductive success through artificial pollination to explore the reproductive characteristics of *V. negundo* var. *heterophylla* for the first time. From the results of fruit set, there is a mixed mating system dominated by outcrossing in *V. negundo* var. *heterophylla* accompanied by self‐compatibility, and it cannot carry out autonomous selfing. Our data show the pollinator‐mediated interaction in the success of reproduction, whereas the effect of anemophily is very weak. And the seed germination rate of inbred line progenies was lower than that of hybrid progenies, which is suspected to be caused by inbreeding depression. The research will provide scientific information for the protection and conservation of *V. negundo* var. *heterophylla* from the point of view of reproduction. In sum, the results are necessary to protect animal vectors in the background of insect decline.

## Graphical abstract

We evaluated reproductive success through artificial pollination to explore the reproductive characteristics of *Vitex negundo* var. *heterophylla* for the first time. From the results of fruit set, there is a mixed mating system dominated by outcrossing in *V. negundo* var. *heterophylla* accompanied by self‐compatibility, and it cannot carry out autonomous selfing.

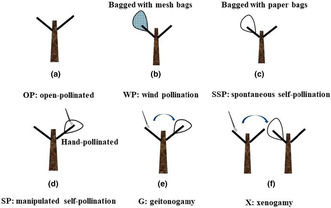

## 1 INTRODUCTION

Plant breeding system is the sum of various physiological and morphological mechanisms that control the relative frequency of outcrossing or self‐crossing, which is an important part of reproductive ecology and a hotspot of evolutionary ecology (Andrus, 1963). On present trends, more and more ecological researches focus on the ecology of plant reproduction (McCallen et al., 2019). As the core of the breeding system, the mating system represents the mating pattern of individuals in a population, which fundamentally answers the question of how individuals mate and the relative frequency of selfing‐outcrossing (Barrett, 1998). The study of plant mating systems and pollination ecology plays a vital role in the cultivation and utilization of plants and the understanding of evolutionary processes. Reproductive ecology, with plant reproduction as its core, organically combines the interaction and mutual adaptation between plant and environment, biological, and abiotic factors (Baucom et al., 2020). Due to the characteristic of fixed growth of plants, pollen must reach the stigma successfully through certain media to achieve reproductive success (Thomson & Page, 2020). Therefore, pollination system is also significant in the process of plant reproduction. Combining flower morphology and pollination system research can objectively understand flower characteristics and their evolutionary driving forces. Research on the reproductive ecology of species is helpful to understand the pollination mechanism of plants, reproductive strategies, and adaptive evolution to the environment. As people pay more attention to the protection of plant diversity, the reproduction characteristics and flowering biological characteristics of more and more species have been revealed step by step (Montagna et al., 2018; Pool‐Chale et al., 2018; Roccotiello et al., 2009; Yamasaki & Sakai, 2013).

Although completely outcrossing or completely selfing mating systems are generally regarded as a stable reproduction strategy, there are few plant groups that are absolutely outcrossing or selfing in nature (Goodwillie et al., 2005). Both models have their own advantages and disadvantages. Selfing will reduce the genetic diversity of individuals and the fitness of offspring due to the expression of harmful traits with higher gene homozygosity (Takebayashi & Morrell, 2001). Outcrossing can accumulate higher levels of genetic variation, but it must be at the cost of increasing investment in reproductive resources (reflected in increasing the attraction of zoophily; Barrett, 1998; Prasifka et al., 2018). Therefore, in order to weigh the pros and cons of selfing and outcrossing, most plants choose a mixed mating system model (Goodwillie et al., 2005). Mating between self and closely related individuals is called inbreeding, and the degree of inbreeding between individuals varies due to differences in genetic history (Schoen & Baldwin, 2022). When plants cannot avoid being selfing due to various mechanisms, inbreeding depression becomes a selective force that regulates the mating dynamics of the population (Han et al., 2021; Husband & Schemske, 1996), which contributes to the evolution and maintenance of the mating system that plays an important part.

Flowers of *Vitex* species are attractive to diverse pollinators, including butterflies, honeybees, and bumblebees (Ashoke & Sudhendu, 2012; Jain, 2013; Murren et al., 2014). The flowers of *Vitex* species are usually hermaphroditic (Schmidt, 2000). Sinebou et al. (2016) had studied the reproductive ecological characteristics of *Vitex doniana* (the endangered African tree species), that this species had hermaphrodite flowers, as well as were adichogamy. A variety of insects of *Hymenoptera* were effective pollinators for *Vitex doniana*, and there was no obstacle for seed germination in the offspring of flower visitors. *Vitex rotundifolia* was found to be capable of both zoogamy and agamogenesis, with strong invasive potential, and pollinator activity directly affecting fruit set and seed setting rate, which may be the mechanism leading to the current successful sexual reproduction (Murren et al., 2014).

Compared with other species in the same genus, *V. negundo* L. var. *heterophylla* exhibited a shorter single flowering period of only 1–2 days, and the previous flower was usually withered when the next flower opened, which greatly reduced the occurrence of geitonogamy (Sun et al., 2020). They also observed in their study that the single flowering period of *V. negundo* L. var. *heterophylla* was indeed prolonged by rainy weather (Sun et al., 2020), and the same phenomenon was observed in *Vitex doniana* (Sinebou et al., 2016). The above mechanisms were beneficial for plants to increase the time and probability of entomophily in the absence of pollinators and was considered to be for ensure reproductive success. The breeding system of plants usually consists of two parts: the development of biological characteristics and the study of mating systems. In the early stage, we have evaluated the natural outcrossing rate of *V. negundo* var. *heterophylla* through genetic markers (SSR) and hybridization index, etc., so as to evaluate the reproductive success and explore the reproductive strategy (Sun et al., 2020).

However, the characteristics of the mating system (self‐breeding and outcrossing strategies between populations and individuals) of *V. negundo* var. *heterophylla* remain unclear, so we mainly want to explore the mating system of *V. negundo* L. var. *heterophylla* through artificial pollination in the later stage. Global pollinator decline and land‐use change may lead to pollination limitation (Goulson et al., 2015). In animal‐pollinated plants, the intensity of interactions with pollinators can be quantified by the degree of pollen limitation of female fitness, that is, the extent to which seed production is limited by insufficient pollination (Trunschke et al., 2017). Manual pollination as a valuable tool can improve pollination control.

In recent years, research on *V. negundo* var. *heterophylla* has mostly focused on environmental adaptation, chemical composition, clinical application, and seed germination (Sun et al., 2020). *Vitex negundo* L. var. *heterophylla* is a common water and soil conservation shrub in northern mountainous and hilly areas and an important nectar plant in China especially in summer (Du et al., 2010). Recent pharmacological studies of *V. negundo* L. var. *heterophylla* had shown a variety of its activities, such as the antioxidative effect (Hu et al., 2017), the antidiabetic effect (Djimabi et al., 2022), the anti‐inflammatory (Xu et al., 2019), and the wound‐healing activities (Han et al., 2008). However, there are few studies on the mating system of *V. negundo* var. *heterophylla* (Yan et al., 2023). Reddy and Reddi found that the flowers of *Vitex negundo* (Lamiaceae) were homogamous, herkogamy, self‐compatible and exhibited a facultative xenogamous breeding system. The scented flowers of *Vitex negundo* (Verbenaceae) were studied to be hermaphrodite and pollinated by entomophily (Reddy & Reddi, 1994). Kumar et al. (2017) carried out a detailed observation of the breeding system of *Vitex chinensis* in India, and the results were similar to the previous studies. The flowers of *Vitex chinensis were* homogamous, herkogamy, self‐compatible and exhibited a facultative xenogamous breeding system. Flowers of *Vitex negundo* L. (Lamiaceae) were found to be self‐compatible and promote xenogamy with narrow chance of spontaneous selfing in nature. (Khan et al., 2021). The RAPD and cpDNA (chloroplast DNA) molecular markers were used to study the genetic structure of 10 populations of *Vitex negundo* (Verbenaceae) in two regions along the Three Gorges River (Zhang et al., 2007). At present, studies on the genetic diversity of *V. negundo* var. *heterophylla* are new but relatively few. Liu et al. (2018) combined functional traits with two molecular markers, which were MSAP (methylation sensitive amplification polymorphism) and AFLP, to analyze phenotypic, genetic, and epigenetic variation in natural populations of *V. negundo* var. *heterophylla* in different habitats. And the results showed that there were significant relationships between epigenetics and genetic variation, epigenetics, and phenotypic variation. Liu et al. (2019) have developed 14 pairs of expressed sequence tags and simple repeat sequence (EST‐SSR) marker primers for this species, which provided useful resources for the study of reproduction and genetic ecology of *V. negundo* var. *heterophylla*. Therefore, there are not many studies on the reproductive ecological characteristics of *V. negundo* var. *heterophylla* at present. Our study plans to conduct a standardized understanding of reproduction information such as the mating system of *V. negundo* var. *heterophylla*, which is implemented through artificial pollination experiments.

In order to clarify the reproduction strategy of *V. negundo* var. *heterophylla*, different kinds of artificial pollination were conducted in the present study. Artificial pollination refers to the artificial transfer of plant pollen to the stigmata in order to improve seed yield, or directional change of plant species. Four indicators of fruit setting rate, fruit weight, germination rate, and germination energy were detected to compare the quality of the progeny of different artificial pollination to evaluate the success of their reproduction. This study makes up for the lack of research on the breeding system of *V. negundo* var. *heterophylla* in China and even in the world and also provides a theoretical background and technical reference for the subsequent research on the pollination biology and the mating systems of relatives of this species.

## 2 MATERIALS AND METHODS

### 2.1 Study site

The study was conducted in a wild population of *V. negundo* var. *heterophylla* (30 × 30 m, population density of 5.3 plants per 100 m^2^) in Qiangu Mountain (36°49′ N, 120°73′ E, 210–220 m above sea level) in Qingdao, Shandong Province, China. The study area belongs to the warm temperate monsoon continental climate and also has significant maritime climate characteristics, which is characterized by humid air, abundant rainfall, moderate temperature, and four distinct seasons. The hottest month is August, with an average temperature of 25.3°C; the coldest month is January, with an average temperature of minus 0.5°C. The average annual temperature is about 12°C, and an average annual rainfall of about 662 mm. According to our measurements, the soil pH was 5.3, and the content of total nitrogen, total phosphorus, and organic matter in soil during the summer were 0.15 (g/kg), 0.24 (g/kg), and 50.8 (g/kg), respectively. In our study sites, *V. negundo* var. *heterophylla* was identified as the dominant species (1–20 cm in diameter) in the study area, and the subdominant species was *Ziziphus jujuba* var. *spinosa* (Rhamnaceae).

### 2.2 Greenhouse germination experiment

**FIGURE 1:**
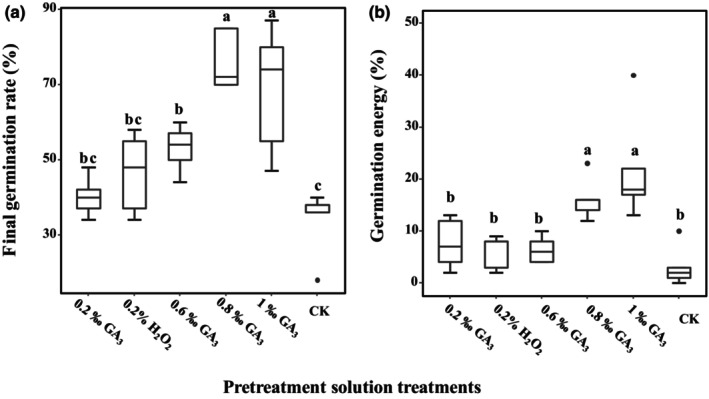
Final germination rate (a) and germination energy (b) after pre‐treatment solution treatments in *Vitex negundo* var. *heterophylla*. Different letters are significantly different at *p* < .05.

Since our knowledge of the most appropriate pre‐treatment for seed germination of *V. negundo* var. *heterophylla* is unknown, we carried out pre‐experiments. The mature seeds of *V. negundo* var. *heterophylla* were collected from 10 naturally distributed plants (ground diameter > 10 cm, growing evenly) of the study site in September 2017. After collection, the fruit stalks and persistent calyxes were rubbed off, then these seeds were stored in a refrigerator at 4°C. Greenhouse germination experiment was carried out from November to December 2017. The seeds were soaked for 24 h with gibberellin acid (GA_3_) of different concentrations containing zero (CK), 0.2 ‰, 0.6 ‰, 0.8 ‰, 1.0 ‰, and 0.2% peroxide hydrogen (H_2_O_2_). The 9 cm Petri dishes with moist double‐decked filter papers were used as germination beds, 20 seeds were placed uniformly in each dish, and 30 dishes (six pre‐treatments × five replicates) were kept in an incubator at 25°C, 60% relative humidity, 14 h light and 10 h darkness for 20 days. The filter papers on the dishes were kept moist throughout the experiment. Seeds were considered germinated at the emergence of the radicle (Bewley & Black, 1994), and without new seed germination for seven consecutive days was marked as the end of the test. Seed germination was recorded daily and expressed as a % percentage of the total number of tested seeds (germination percentage). The final germination rate (germination percentage at 20 days after the test started) and germination energy (germination percentage at 8 days after the test started) were calculated. Trough studying the effect of different concentration and type of solution on the seed growth, the optimum pre‐treatment solution was obtained in the greenhouse germination process. The results of this part of the experiment are shown in Figure 1.

### 2.3 Artificial pollination experiment

We conducted the hand‐pollinated experiment on 10 individuals (growing evenly) in June to July 2019 to evaluate mating system. Since the single flower of *V. negundo* var. *heterophylla* was small, the inflorescence was used as a unit for treatment. On each plant, 18 inflorescences (6 treatments × 3 replicates) were randomly marked, each single flower in each inflorescence was treated the same way. Note that in the selection of experimental subjects, the selected inflorescence was basically the same in terms of length, height, and other dimensions, thus reducing the possibility caused by this problem. Stigma receptivity was examined prior to pollination. We used the hydrogen peroxide method (Dafni, 1992) to test the stigma receptivity. Stigmas were collected at 0, 3, 6, 9, 12, and 24 h after flowering, and 10 stigmas were randomly collected at the observation site at each stage. The stigma was immersed in a plastic bottle cap containing 3% H_2_O_2_ reaction solution, and the appearance and number of bubbles on the stigma were observed under a magnifying glass (11×). The more bubbles, the stronger the stigma receptiveness. The stigma morphology at different stages after flowering was also recorded. For details, please refer to the article (Sun et al., 2020).

**FIGURE 2:**
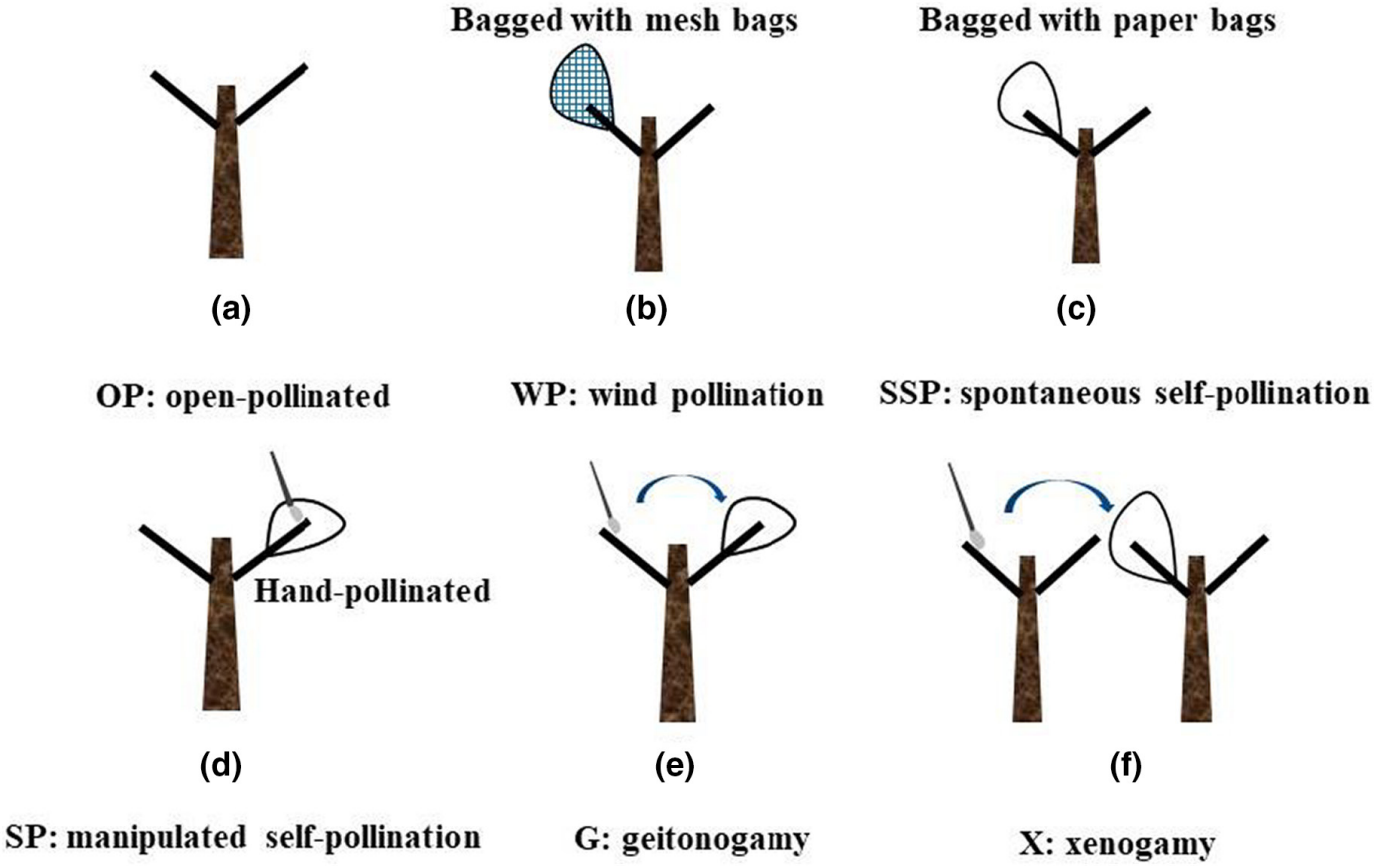
Different artificial pollination treatments in *Vitex negundo* var. *heterophylla*. Open pollination (OP), anemophily (WP), autonomous selfing (SSP), autogamy (SP), geitonogamy (G), xenogamy (X). (a) OP: open pollination, detecting pollination under natural conditions and as comparison; (b) WP: anemophily, detasseling and netting to detect the degree of anemophily; (c) SSP: autonomous selfing; setting the paper bag, except for other operations, to check whether it can be spontaneously pollinated; (d) SP: autogamy; setting paper bags and performing flush pollination; (e) G: geitonogamy, detasseling paper bags and cross‐pollinating the same plant; (f) X: xenogamy, detasseling paper bags and allogamy of different plants.

Pollination treatments including: (1) OP (Figure 2a): open‐pollinated by pollinators; (2) WP (Figure 2b): emasculated and bagged with mesh bags to detect wind pollination; (3) SSP (Figure 2c): bagged with paper bags and left unmanipulated to detect spontaneous self‐pollination; (4) SP (Figure 2d): hand‐pollinated with pollen from the same flower using a small brush and bagged to detect manipulated self‐pollination; (5) G (Figure 2e): emasculated, hand‐pollinated with pollen from another flower of the same tree and bagged to detect geitonogamy; (6) X (Figure 2f): emasculated, hand‐pollinated with pollen from another tree and bagged to detect xenogamy. The bagging was carried out at bud phase and all hand‐pollinated flowers were bagged with paper pollination bags to exclude pollinators. (4) and (5) were to detect the degree of self‐compatibility and (6) was to detect the degree of hybridization. The number of treated flowers per labeled inflorescence was recorded every day. Nine thousand seventy four flowers of *V. negundo* var. *heterophylla* were treated in total. The number of flowers per inflorescence under different pollination treatments are in Table S2.

Before the six treatments, stigmatic receptivity was checked to ensure that the selected branches were healthy and normally developed. Bagging was carried out at the bud stage. According to the study (Sun et al., 2020), the best pollination time was determined to be from 10:00 am to 12:00 am in view of the pollen viability and stigmatic receptivity at different stages after flowering, and with the help of tweezers, the anther directly contacted the stigma for pollination. The phenological developmental stage of the flower at pollination was during the anthesis. Then we followed up the post‐pollination development and subsequently collected the progeny of the treated inflorescences for subsequent fruit weight determination and germination experiments.

### 2.4 Evaluation of reproductive success strategies

The progeny of the treated inflorescence was collected, and the subsequent fruit weight determination and germination experiment were conducted. Four indicators of fruit setting rate, fruit weight, germination rate, and germination energy were used to compare the quality of the progeny of different pollination treatments to evaluate the success of their reproduction.

#### 2.4.1 Fruit setting rate and mean fruit weight

During the peak flowering period from June 26 to July 18, 2019, all target branches were marked and the number of flowers on each branch was calculated. Then we collected the fruits of six groups (OP, WP, SSP, SP, G, X) of *V. negundo* var. *heterophylla* and divided the number of fruits by the number of flowers to get the fruit setting rate.

#### 2.4.2 Average fruit weight

We randomly selected 100 ripe fruits from the four treatments of OP, SP, G, and X, then weighed them. We calculated the average fruit weight of a single kernel. The above operation needed to be repeated five times for each group.

#### 2.4.3 Seed germination rate and germination energy

The mature seeds of *V. negundo* var. *heterophylla* were collected from 10 naturally distributed plants (ground diameter > 10 cm, growing evenly) of the study site in September 2019. After collection, the fruit stalks and persistent calyxes were rubbed off, and these seeds were stored in a refrigerator at 4°C. Greenhouse germination experiment was conducted on the seeds of OP, SP, G, and X treatments in October 2019. The seeds were soaked with gibberellin solution of 1000 mg·L^−1^ for 24 h and were put on a germination bed of a 9 cm petri dish with 2 layers of moist filter paper. We placed 25 seeds in each dish evenly and there were five replicates per treatment to reduce the error. These experiments were carried out in an incubator with a relative humidity of 60%, a temperature of 25°C, 14 h of light and 10 h of darkness. The filter papers on the dishes were kept moist throughout the experiment. Seeds were considered germinated at the emergence of the radicle (Bewley & Black, 1994) and without new seed germination for seven consecutive days was marked as the end of the test. Seed germination was recorded daily and expressed as a % percentage of the total number of tested seeds (germination percentage). The final germination rate (germination percentage at 20 days after the test started) and germination energy (germination percentage at 8 days after the test started) were calculated. The germination rate (GR) and germination energy (GE) were calculated according to Formulas (1 and 2).

(1) $$\mathrm{GR}\;\left(\%\right)=\left(n/N\right)\times 100\%$$

where GR was the germination rate; *n* was the number of germinated seeds; *N* was the total number of tested seeds.

(2) $$\mathrm{GE}\;\left(\%\right)=\left(m/N\right)\times 100\%$$

GE was the germination energy; *m* was the number of germinated seeds when the number of germinated seeds reached the highest peak (the first 8 days in present study); *N* was the total number of tested seeds. Seed quality evaluation of pollination treatment in Qiangu Mountain was in Table S3.

### 2.5 Data analyses

The data were first tested using chi‐squared and normality tests, and no log transformation or square root transformation was used. First, one‐way ANOVA was used to test the final germination rate and germination energy of the pre‐experimental part, and then Least Significant Difference (LSD) method was used for multiple comparisons, and the significance threshold was set at 0.05. One‐way ANOVA was employed to evaluate the reproductive success indicators of different pollination treatments, and post hoc comparison was conducted based on Fisher's LSD with a significance threshold set at 0.05. The same data analysis method was also used to examine the fruit set, mean fruit mass, final germination rate, and germination energy among pollination treatments. The statistical analysis was performed using R 3.4.3 software (R Core Team, 2017; R Development Core Team, 2017). The data was then visualized using the ggplot2 package (Wickham, 2016) in R.

## 3 RESULTS

### 3.1 Pre‐experimental results

By performing germination tests after immersion in different concentrations of GA_3_ and H_2_O_2_ solutions, we found that the final germination rate (Figure 1a) and germination potential (Figure 1b) of the seeds of *V. negundo* var. *heterophylla* were greatest after treatment with a GA_3_ pre‐treatment solution at a concentration of 1‰, with the final germination rate of 68.60% (SD = 0.0695) and the germination energy of 22 (SD = 0.1006), and thus in pre‐treatment solution was most favorable for seed germination of *V. negundo* var. *heterophylla*.

### 3.2 Fruit setting rate

**FIGURE 3:**
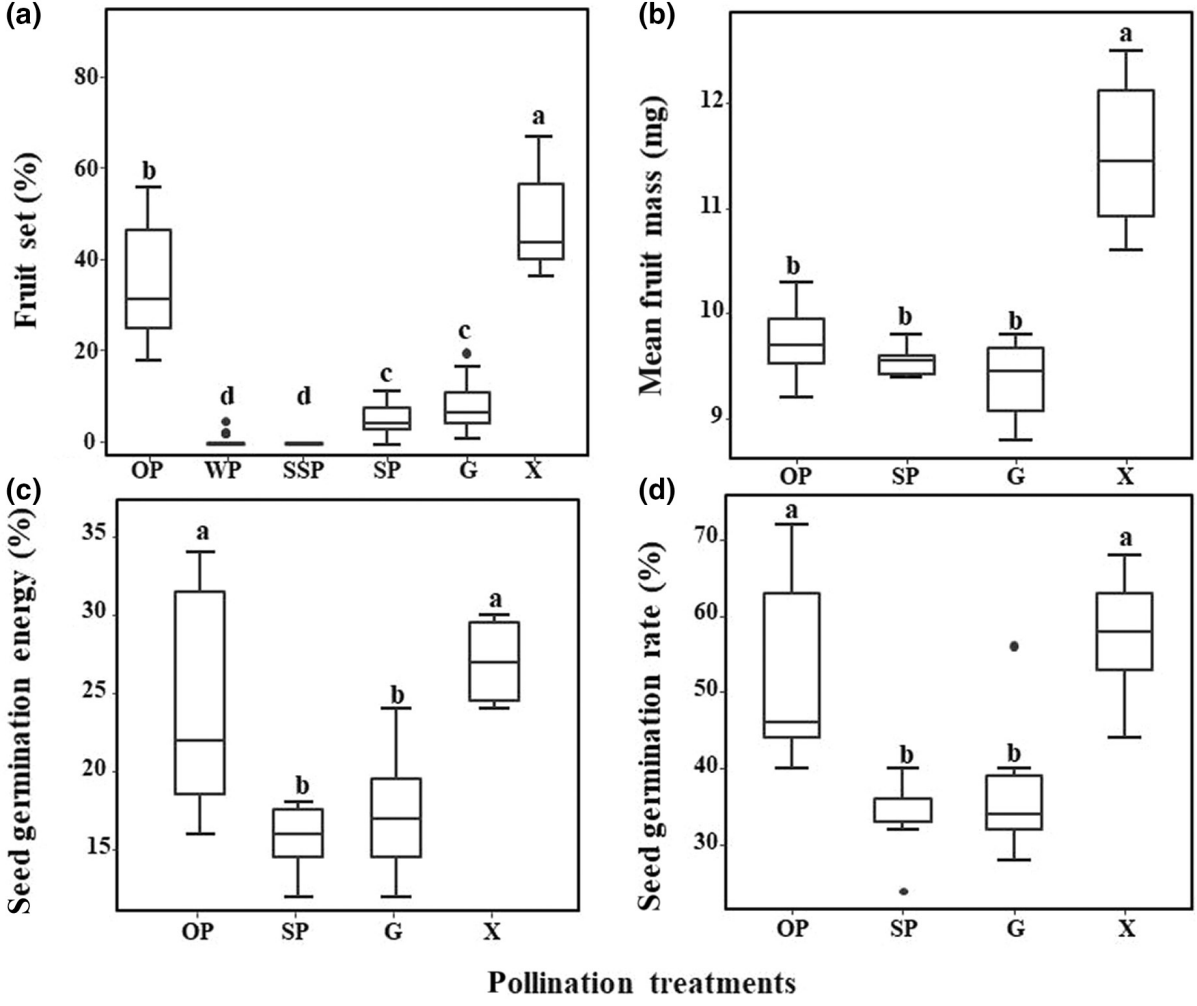
Fruit set (a, *n* = 20), mean fruit mass (b, *n* = 5), seed germination energy (c, *n* = 5), and seed germination rate (d, *n* = 5) after different pollination treatments in *Vitex negundo* var. *heterophylla*. Pollination treatments: open pollination (OP), anemophily (WP), autonomous selfing (SSP), autogamy (SP), geitonogamy (G), and xenogamy (X). Different letters are significantly different at *p* < .05.

Taking into account the man‐made and natural damage to the processed inflorescences during the experiment, we selected 20 healthy inflorescences from the harvest samples for fruit setting statistics (Figure 3).

Through a one‐way analysis of variance, it was found that the fruit setting rate was significantly correlated with pollination methods (*F* = 182.2, *p* < .01; Figure 3a). Xenogamy (X, 47.0%, SD = 0.0447) can significantly increase the fruit setting rate than natural conditions (OP, 35.8%, SD = 0.0842), while the fruit setting rate under autogamy (SP, 5.7%, SD = 0.0124) and geitonogamy (G, 8.3%, SD = 0.0321) was significantly lower than that under open pollination (OP, 35.8%, SD = 0.0324). The results revealed that *V. negundo* L. var. *heterophylla* has self‐compatibility and can successfully produce offspring through selfing, but there was inbreeding depression during the seed maturation process.

The anemophily (WP, 0.5%, SD = 0.0255, Figure 3a) was also observed which can produce offspring, although the fruit setting rate was very low. It was clear that *V. negundo* L. var. *heterophylla* cannot carry out spontaneously autogamy (SSP, 0%, SD = 0, Figure 3a), and the fruit setting rate under this pollination mode was always 0 and there was no apomixes. Only one of the four ovules of *V. negundo* L. var. *heterophylla* can fully develop and bear fruit, and the fruit setting rate is constantly at 25%.

### 3.3 Average fruit weight

Due to the limited number of progeny collected, we only evaluate the progeny of OP, SP, X, and G for further reproductive success evaluation.

Through a one‐way analysis of variance, it was found that the average fruit weight was significantly correlated with pollination methods (*F* = 27.65, *p* < .01; Figure 3b). The average fruit of xenogamy (X, 11.7 mg, SD = 0.7036) was significantly higher than the other three pollination methods (OP: 9.8 mg, SD = 0.4278; SP: 9.5 mg, SD = 0.1673; G: 9.5 mg, SD = 0.3271). The average fruit weight was not significantly different among the three methods of pollination of OP, SP, and G. And the average fruit weight of autogamy and geitonogamy were similar, which were not significantly different with open pollination.

### 3.4 Seed germination rate and germination energy

Both of seed germination energy (*F* = 7.508, *p* < .01; Figure 3c) and germination rate (*F* = 7.931, *p* < .01; Figure 3d) had a significant correlation with pollination methods. And the above two indicators showed the same trend among different pollination modes.

The germination energy (27.6%, SD = 0.0261) of xenogamy (X) was significantly higher than autogamy (SP, 15.2%, SD = 0.0521) and geitonogamy (G, 16.0%, SD = 0.0456), and there was no significant difference between open pollination (OP, 24.4%, SD = 0.0137). Similarly, the seed germination rate of xenogamy (X, 60.0%, SD = 0.0632) was significantly higher than autogamy (SP, 32.8%, SD = 0.0167) and geitonogamy (G, 33.6%, SD = 0.0456), and there was no significant difference between open pollination (OP, 55.2%, SD = 0.0137). There was no statistical difference in seed germination energy and germination rate between autogamy (SP) and geitonogamy(G). Intra‐group degrees of freedom for seed quality evaluation of different pollination treatments were in Table S1.

## 4 DISCUSSION

Through controlled pollination experiments, it was found that *V. negundo* L. var. *heterophylla* can produce mature fruits under both outcrossing (the highest fruit setting rate) and selfing strategies, indicating that *V. negundo* L. var. *heterophylla* presents a mixed mating system with mainly outcrossing and self‐compatibility. It is consistent with the conclusion of Sun et al. (2020) based on the hybridization index and pollen ovule ratio.

The maintenance of the mixed mating system is the result of continuous evolution under the selective pressure of outcrossing and selfing. It can not only adapt to the plant itself and environmental conditions but also ensure the successful reproduction of plants under environmental mutations. The dire state of insect biodiversity in the world due to the serious decline in insect diversity due to the widespread use of agricultural pesticides, as almost half of species are rapidly declining and one‐third are threatened with extinction (Sánchez‐Bayo & Wyckhuys, 2019). When outcrossing cannot be carried out under the conditions of habitat change or restricted pollination, choosing selfing can provide a certain amount of protection for the reproduction of plants (Lloyd, 1992). Other species of the genus *Vitex* have also been reported to have mixed mating systems in which selfing and outcrossing coexist, such as *V. lucens* (Barrell et al., 1997), *V. doniana* (Sinebou et al., 2016), and *V. rotundifolia* (Murren et al., 2014) and *V. fischeri* (Lengkeek et al., 2006).

In present artificial pollination experiments, we also found that autonomous selfing cannot happen to *V. negundo* L. var. *heterophylla*, and there is no apomixis. Anemophily (WP) can only produce very few offspring (fruit setting rate is 0.5%), and the effect on allogamy is minimal. Therefore, despite the coexistence of entomophily and anemophily flower morphology, this species eventually reproduces successfully. It still depends entirely on the service level of zoophily. In order to adapt to insect pollination, *V. negundo* L. var. *heterophylla* also exhibits a series of special floral morphology: (1) In most *Vitex* species, the stamens and styles are located near the upper lip, conforming to the esophageal type of flower described by van der Pijl (1972). *Vitex negundo* L. var. *heterophylla* has a bilabial flower that is symmetrical, and the lower lip of the bilabial corolla is flat and broad, which can be used as a comfortable platform for insects to forage (Sun et al., 2020). (2) The terminal panicle tightly gathers the flowers together, reducing the time for insects to fly and search for nectar or pollen, which is conducive to the maximum use of their own energy for foraging; (3) the flower secretes nectar and releases fragrance during the flowering process to attract insects (van der Pijl, 1972). Due to the limitations of various conditions in the study site, we did not record pollinator observation, visit frequency or other indicators. During the study, we only observed the flower visitors of *V. negundo* L. var. *heterophylla* in general. Therefore, no data are currently available to support a comprehensive understanding of the species' pollinators and their flower‐visiting behavior. Then we will further study the type, number, frequency, stay time, and effective pollinators of flower visitors. In addition, it is necessary to pay more attention to the pollinator‐mediated floral evolution of some herkogamy in *Vitex* sinensis population.

A similar situation also occurred in *Thalictrum pubescens*, an ambophilous (both zoophily and anemophily) species that probably represents a transitional state in the evolution of anemophily. And the ambophily was also studied in *Olea ferruginea*, over a period of 3 years. It was an andromonoecious and outcrossing tree species of the olive complex. The species was predominately pollinated by wind because it showed fruit set even when the insect pollinators were excluded (Khan et al., 2022). *Mallotus* spp. (Euphorbiaceae) *japonicus* and *Mallotus wrayi* exhibited floral characteristics that were adapted to both wind and insect pollination, ambophily may be actively maintained in the two species at the study sites and perhaps elsewhere (Yamasaki & Sakai, 2013). Other studies had shown that the flowers of *Crateva adansonii* (Capparaceae) exhibited traits conducive to a mixture of wind and insect pollination (ambophily). Although a variety of insects visited the flowers, they were ineffective in pollinating. Nevertheless, active foraging by the honeybees (*Apis dorsata, A. mellifera* and *A. cerana indica*) facilitated enhanced pollen dispersal in the air and resulted in indirect pollination by wind (Mangla & Tandon, 2011). While zoophily mediate, the advantage of anemophily will be weakened (Labouche et al., 2017). In addition, the seed setting rate of *V. negundo* L. var. *heterophylla* is a constantly 25%, which means that only one of the four ovules can fully develop and bear fruit, and the other three will be aborted due to late zygotic competition. The same phenomenon is found in *V. fischeri* (Lengkeek et al., 2006) and *V. Doniana* (Sinebou et al., 2016) is also reported. This is not due to pollen limitation, but may be related to resource allocation. Research by Zoeller et al. (2017) shows that artificial pollination of two species of geoflorus *Protea* species (Proteaceae) has not improved their seed setting rate, and resources are limited.

Inbreeding depression was defined as the reduced fitness of selfing progeny relative to outcrossed progeny, it was a frequent phenomenon in plants that can be expressed at various stages in the life cycle (Charlesworth & Charlesworth, 1987; Husband & Schemske, 1996). It was a common experimental technique to judge the degree of inbreeding depression by germination rate and germination energy. Ramsey et al, germinated selfing and outcrossed seeds from the same *Blandfordia grandiflora* plants and estimated inbreeding depression in germination under benign laboratory conditions and adverse field conditions. They found that 60% of *Blandfordia grandiflora* expressed inbreeding depression in the laboratory, whereas in the field all plants expressed inbreeding depression. A similar example would be *Echium wildpretii* (Boraginaceae) on Tenerife, Canary Islands. Researchers investigated the fitness of progeny from experimental self‐ and cross‐pollination in eight populations of different size of *E. wildpretii*, and found that seed set of open‐ and hand‐outcrossed‐pollinated flowers was higher in large than in small populations, possibly due to more frequent biparental inbreeding in the latter (Sedlacek et al., 2012). Through the study on combining the analysis of floral morphology, behavior of flower visitors, and artificial pollination, the reproductive characteristics of the *Ziziphus jujuba* var. *spinosa* were revealed (Wang et al., 2021). In this study, through artificial pollination experiment, we determined that the inbred progeny of *V. negundo* L. var. *heterophylla* had serious inbreeding decline in early seed setting and germination stage and it was difficult to produce seeds.

We conclude that there was still inbreeding depression during the process of *V. negundo* L. var. *heterophylla* seedlings. The inbreeding depression occurs in various stages of life history of an individual to varying degrees (Van Etten et al., 2021; Voillemot & Pannell, 2017). Species with different mating systems may have different stages and degrees of inbreeding depression (Van Etten et al., 2021). Inbreeding depression of some species is mainly manifested in the early stage of seed setting and germination, while others are mainly manifested in the later stage of survival and growth (Sinebou et al., 2016). When we evaluated the quality of the progeny of *V. negundo* L. var. *heterophylla* under different pollination treatments, we found that the fruit weight of *V. negundo* L. var. *heterophylla* outcrossing progeny was significantly higher than that of the selfing progeny. Similarly, in the process of seed germination, the seed germination rate and germination energy of outcrossing progeny were significantly higher than that of selfing progeny, which indicated that *V. negundo* L. var. *heterophylla* had different degrees of inbreeding depression during seed maturation and germination.

From the above results, it can be inferred: the *V. negundo* L. var. *heterophylla* is mainly autogamy, as well as be allogamy; it has the phenomenon of selfing but cannot finish the autonomous selfing. The *V. negundo* L. var. *heterophylla* depends on entomophily for their pollination; The inbreeding progeny of *V. negundo* L. var. *heterophylla* had serious inbreeding depression at early seed setting and germination stage. It is worth to be discussed that in this study, only the general observation of the visiting population of *V. negundo* L. var. *heterophylla* was carried out during the experiment, and there is no data to support a comprehensive understanding of the species and number of pollinators and their visiting behavior. Further studies are needed to investigate the types and numbers of visitors, visiting frequency, staying time, and effective pollinators. Whether the herkogamy in flower evolution of *V. negundo* L. var. *heterophylla* is mediated by pollinators needs to be further investigated by various experimental methods.

In this study, we conducted artificial pollination experiments to further understand the mating system and other reproductive strategies of *V. negundo* L. var. *heterophylla*. Considering the *V. negundo* L. var. *heterophylla* has the function of slope soil and water conservation, the mating strategy revealed by results will help to protect and effectively use the plant resources and provide a scientific basis for the breeding of wild varieties.

## AUTHOR CONTRIBUTIONS

**Qing Zhang:** Conceptualization (equal); investigation (lead); methodology (equal); writing – original draft (lead). **Jilin Zhang:** Investigation (supporting). **Xiaohan Sun:** Investigation (supporting). **Feng Wang:** Conceptualization (supporting); methodology (supporting). **Renqing Wang:** Conceptualization (equal); methodology (equal); writing – review and editing (equal). **Hui Wang:** Conceptualization (equal); methodology (equal); writing – review and editing (lead). **Peiming Zheng:** Conceptualization (equal); methodology (equal); writing – review and editing (equal).

## CONFLICT OF INTEREST STATEMENT

The authors have declared that no competing interests exist.

## Supporting information


Appendix S1.

## Data Availability

The data that support the findings of this study are openly available in Dryad. It contains the raw data of germination rate experiment, seed quality evaluation experiment, pre‐experiment, and code for data analysis. The details are as follows: Dryad https://doi.org/10.5061/dryad.6t1g1jx57 or https://datadryad.org/stash/share/Msigcqh53G5rLuiu_4HySPH4aFCcXclNW‐tI8o14vuQ.
