# Correction to “Mating strategies of *Vitex negundo* L. var. *heterophylla* (Franch.) Rehder (Lamiaceae): A mixed mating system with inbreeding depression”

**DOI:** 10.1002/ece3.11165

**Published:** 2024-03-17

Zhang, Q., Zhang, J., Sun, X., Wang, F., Wang, R., Wang, H., & Zheng, P. (2024). Mating strategies of *Vitex negundo* L. var. *heterophylla* (Franch.) Rehder (Lamiaceae): A mixed mating system with inbreeding depression. *Ecology and Evolution*, 14, e10927. https://doi.org/10.1002/ece3.10927.

In the first page of the contribution of the authors, the “All authors are contributed equally to this work” was incorrect.

This sentence “All authors are contributed equally to this work” should be deleted.

We apologize for this error.

