# Supplementary material for: Mating strategies of Vitex negundo L. var. heterophylla (Franch.) Rehder (Lamiaceae): A mixed mating system with inbreeding depression
Source: Ecol Evol. 2024 Feb 26;14(2):e10927. doi: 10.1002/ece3.10927 (PMC10897527; doi:10.1002/ece3.10927)
Supplement: Supplementary file 1 — Appendix S1. [file ECE3-14-e10927-s001.docx]

**Supplementary Material**

Table S1: Intra-group degrees of freedom for seed quality evaluation of different pollination treatments.

| Freedoms within the group | OP | SP | G | X | SSP | WP |
| --- | --- | --- | --- | --- | --- | --- |
| fruit set (Fig3 a) | 2397 | 1835 | 1989 | 1778 | 1069 | 1020 |
| mean fruit mass (Fig3 b) | 753 | 109 | 151 | 837 | / | / |
| seed germination energy (Fig3 c) | 496 | 496 | 496 | 496 | / | / |
| seed germination ratio (Fig3 d) | 496 | 496 | 496 | 496 | / | / |

Open pollination (OP), anemophily (WP), autonomous selfing (SSP), autogamy (SP), geitonogamy (G), xenogamy (X).

Table S2: Number of flowers per inflorescence under different pollination treatments.

| Inflorescence numbering | N treated | SP treated | G treated | X treated | SSP treated | WP treated |
| --- | --- | --- | --- | --- | --- | --- |
| 1 | 119 | 71 | 77 | 70 | 44 | 48 |
| 2 | 101 | 78 | 67 | 56 | 32 | 58 |
| 3 | 45 | 60 | 87 | 70 | 38 | 52 |
| 4 | 79 | 41 | 59 | 57 | 21 | 24 |
| 5 | 53 | 58 | 53 | 63 | 56 | 54 |
| 6 | 68 | 58 | 98 | 46 | 43 | 58 |
| 7 | 53 | 56 | 50 | 78 | 29 | 45 |
| 8 | 131 | 86 | 66 | 69 | 31 | 45 |
| 9 | 96 | 72 | 67 | 77 | 39 | 30 |
| 10 | 109 | 58 | 54 | 49 | 41 | 26 |
| 11 | 56 | 107 | 92 | 33 | 52 | 16 |
| 12 | 65 | 86 | 66 | 50 | 46 | 32 |
| 13 | 72 | 74 | 57 | 64 | 28 | 30 |
| 14 | 129 | 52 | 64 | 55 | 40 | 19 |
| 15 | 93 | 44 | 40 | 65 | 35 | 51 |
| 16 | 115 | 66 | 84 | 85 | 23 | 35 |
| 17 | 86 | 67 | 84 | 80 | 29 | 21 |
| 18 | 59 | 38 | 63 | 65 | 33 | 39 |
| 19 | 92 | 45 | 82 | 63 | 38 | 18 |
| 20 | 69 | 49 | 78 | 47 | 45 | 24 |
| 21 | 90 | 54 | 73 | 53 | 39 | 51 |
| 22 | 67 | 87 | 64 | 59 | 51 | 42 |
| 23 | 69 | 48 | 58 | 64 | 47 | 22 |
| 24 | 59 | 65 | 72 | 69 | 28 | 28 |
| 25 | 92 | 55 | 48 | 74 | 32 | 34 |
| 26 | 69 | 73 | 56 | 54 | 24 | 40 |
| 27 | 57 | 45 | 87 | 49 | 31 | 28 |
| 28 | 81 | 59 | 76 | 59 | 41 | 33 |
| 29 | 129 | 89 | 73 | 61 | 39 | 23 |
|  | 2403 | 1841 | 1995 | 1784 | 1075 | 1026 |

Open pollination (OP), anemophily (WP), autonomous selfing (SSP), autogamy (SP), geitonogamy (G), xenogamy (X). The last line is the sum of the number of flowers in each treatment.

Table S3: Seed quality evaluation of pollination treatment in Qiangu Mountain

| Inflorescence numbering | Pollination Trials | Mean fruit mass (mg) | Germination Energy （%） | Germination Rate（%） | SD（Standard Deviation） | SEM（Standard error of mean） | CV（Coefficient of Variance） |  |
| --- | --- | --- | --- | --- | --- | --- | --- | --- |
|  |  |  |  |  |  |  |  |  |
| 1 | OP | 10.3 | 18 | 44 | 0.4278 | 0.1913 | 0.0438 |  |
| 2 | OP | 9.5 | 34 | 68 |  |  |  |  |
| 3 | OP | 10 | 34 | 72 |  |  |  |  |
| 4 | OP | 9.2 | 16 | 48 |  |  |  |  |
| 5 | OP | 9.8 | 20 | 44 |  |  |  |  |
| 1 | SP | 9.4 | 12 | 24 | 0.1673 | 0.0748 | 0.0175 |  |
| 2 | SP | 9.6 | 16 | 36 |  |  |  |  |
| 3 | SP | 9.8 | 14 | 32 |  |  |  |  |
| 4 | SP | 9.4 | 16 | 36 |  |  |  |  |
| 5 | SP | 9.5 | 18 | 36 |  |  |  |  |
| 1 | G | 9.8 | 12 | 32 | 0.3271 | 0.1463 | 0.0345 |  |
| 2 | G | 9.6 | 14 | 28 |  |  |  |  |
| 3 | G | 9.7 | 20 | 32 |  |  |  |  |
| 4 | G | 9.3 | 18 | 36 |  |  |  |  |
| 5 | G | 9 | 16 | 40 |  |  |  |  |
| 1 | X | 12.5 | 28 | 52 | 0.7036 | 0.3146 | 0.0601 |  |
| 2 | X | 11.3 | 24 | 68 |  |  |  |  |
| 3 | X | 10.8 | 26 | 56 |  |  |  |  |
| 4 | X | 11.6 | 30 | 60 |  |  |  |  |
| 5 | X | 12.3 | 30 | 64 |  |  |  |  |

Pollination treatments: open pollination (OP), autogamy (SP), geitonogamy (G), and xenogamy (X).

Table S4: Raw data of seed germination experiment

| Inflorescence | fruit set | G | mature fruits | fruit set | X | mature fruits | fruit set | SSP | mature fruits | fruit set | WP | mature fruits | fruit set |  |
| --- | --- | --- | --- | --- | --- | --- | --- | --- | --- | --- | --- | --- | --- | --- |
|  |  |  |  |  |  |  |  |  |  |  |  |  |  |  |
| 1 | 11.27% | 77 | 7 | 9.09% | 70 | 34 | 48.57% | 44 | 0 | 0 | 48 | 1 | 2.08% |  |
| 2 | 6.41% | 67 | 3 | 4.48% | 56 | 26 | 46.43% | 32 | 0 | 0 | 58 | 0 | 0.00% |  |
| 3 | 8.33% | 87 | 2 | 2.30% | 70 | 26 | 37.14% | 38 | 0 | 0 | 52 | 1 | 1.92% |  |
| 4 | 9.76% | 59 | 10 | 16.95% | 57 | 26 | 45.61% | 21 | 0 | 0 | 24 | 1 | 4.17% |  |
| 5 | 8.62% | 53 | 9 | 16.98% | 63 | 25 | 39.68% | 56 | 0 | 0 | 54 | 0 | 0.00% |  |
| 6 | 3.45% | 98 | 17 | 17.35% | 46 | 26 | 56.52% | 43 | 0 | 0 | 58 | 1 | 1.72% |  |
| 7 | 3.57% | 50 | 7 | 14.00% | 78 | 32 | 41.03% | 29 | 0 | 0 | 45 | 0 | 0.00% |  |
| 8 | 3.49% | 66 | 6 | 9.09% | 69 | 28 | 40.58% | 31 | 0 | 0 | 45 | 0 | 0.00% |  |
| 9 | 8.33% | 67 | 4 | 5.97% | 77 | 35 | 45.45% | 39 | 0 | 0 | 30 | 0 | 0.00% |  |
| 10 | 12.07% | 54 | 3 | 5.56% | 49 | 20 | 40.82% | 41 | 0 | 0 | 26 | 0 | 0.00% |  |
| 11 | 5.61% | 92 | 2 | 2.17% | 33 | 20 | 60.61% | 52 | 0 | 0 | 16 | 0 | 0.00% |  |
| 12 | 3.49% | 66 | 1 | 1.52% | 50 | 22 | 44.00% | 46 | 0 | 0 | 32 | 0 | 0.00% |  |
| 13 | 4.05% | 57 | 4 | 7.02% | 64 | 29 | 45.31% | 28 | 0 | 0 | 30 | 0 | 0.00% |  |
| 14 | 3.85% | 64 | 5 | 7.81% | 55 | 37 | 67.27% | 40 | 0 | 0 | 19 | 0 | 0.00% |  |
| 15 | 6.82% | 40 | 3 | 7.50% | 65 | 36 | 55.38% | 35 | 0 | 0 | 51 | 0 | 0.00% |  |
| 16 | 6.06% | 84 | 9 | 10.71% | 85 | 33 | 38.82% | 23 | 0 | 0 | 35 | 0 | 0.00% |  |
| 17 | 2.99% | 84 | 5 | 5.95% | 80 | 34 | 42.50% | 29 | 0 | 0 | 21 | 0 | 0.00% |  |
| 18 | 0.00% | 63 | 9 | 14.29% | 65 | 27 | 41.54% | 33 | 0 | 0 | 39 | 0 | 0.00% |  |
| 19 | 2.22% | 82 | 2 | 2.44% | 63 | 27 | 42.86% | 38 | 0 | 0 | 18 | 0 | 0.00% |  |
| 20 | 4.08% | 78 | 4 | 5.13% | 47 | 28 | 59.57% | 45 | 0 | 0 | 24 | 0 | 0.00% |  |
| 21 | 7.41% | 73 | 5 | 6.85% | 53 | 25 | 47.17% | 39 | 0 | 0 | 51 | 1 | 1.96% |  |
| 22 | 6.90% | 64 | 3 | 4.69% | 59 | 35 | 59.32% | 51 | 0 | 0 | 42 | 0 | 0.00% |  |
| 23 | 2.08% | 58 | 5 | 8.62% | 64 | 38 | 59.38% | 47 | 0 | 0 | 22 | 0 | 0.00% |  |
| 24 | 3.08% | 72 | 8 | 11.11% | 69 | 39 | 56.52% | 28 | 0 | 0 | 28 | 0 | 0.00% |  |
| 25 | 9.09% | 48 | 3 | 6.25% | 74 | 41 | 55.41% | 32 | 0 | 0 | 34 | 0 | 0.00% |  |
| 26 | 6.85% | 56 | 3 | 5.36% | 54 | 21 | 38.89% | 24 | 0 | 0 | 40 | 1 | 2.50% |  |
| 27 | 6.67% | 87 | 8 | 9.20% | 49 | 19 | 38.78% | 31 | 0 | 0 | 28 | 0 | 0.00% |  |
| 28 | 13.56% | 76 | 5 | 6.58% | 59 | 23 | 38.98% | 41 | 0 | 0 | 33 | 0 | 0.00% |  |
| 29 | 6.74% | 73 | 3 | 4.11% | 61 | 29 | 47.54% | 39 | 0 | 0 | 23 | 1 | 4.35% |  |

Pollination treatments: autonomous selfing (SSP), autogamy (SP), geitonogamy (G), and xenogamy (X).


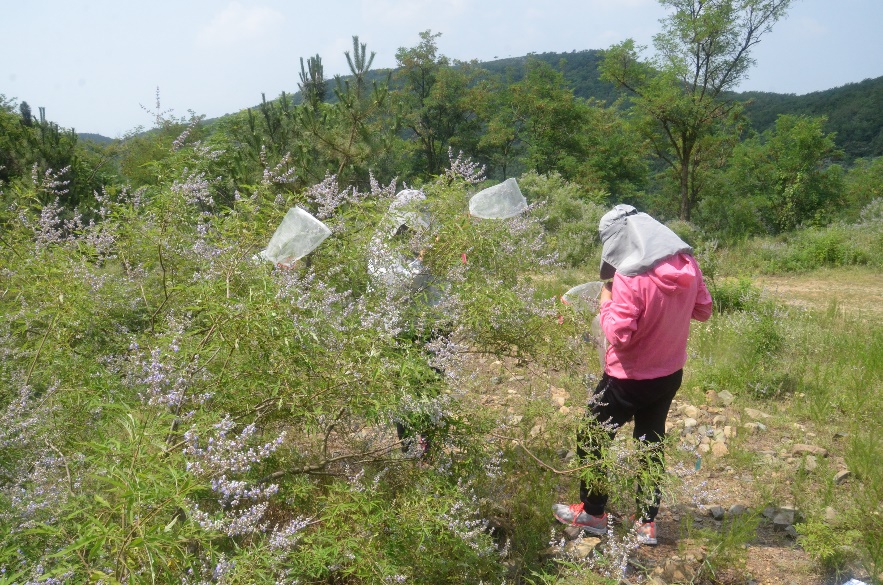

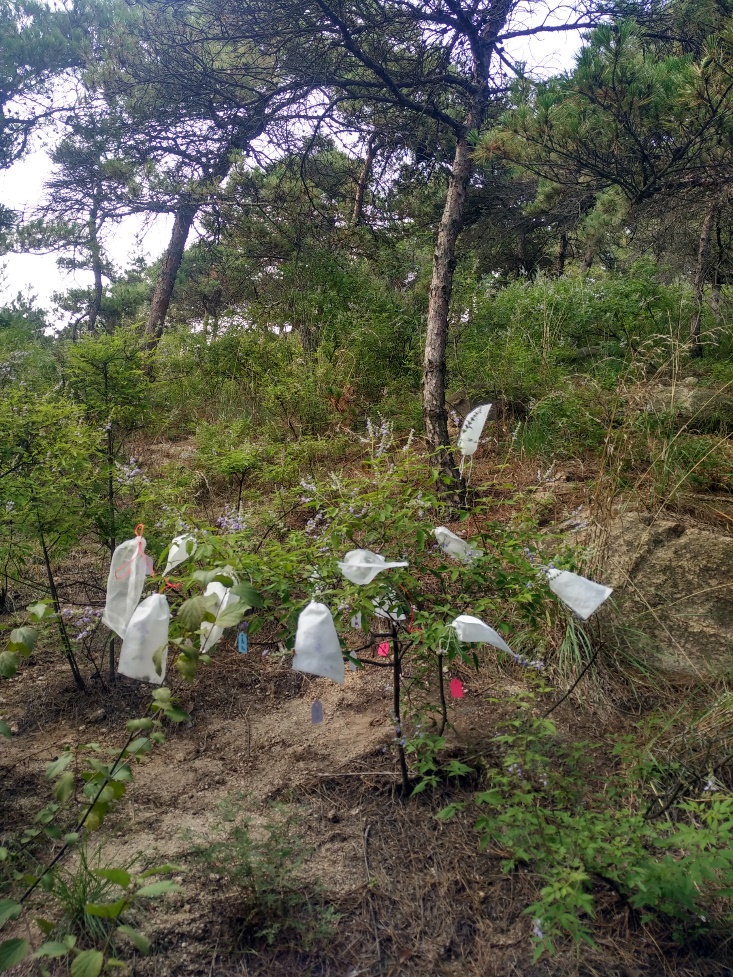
Figure S1: Photographs of the different pollination treatments applied.


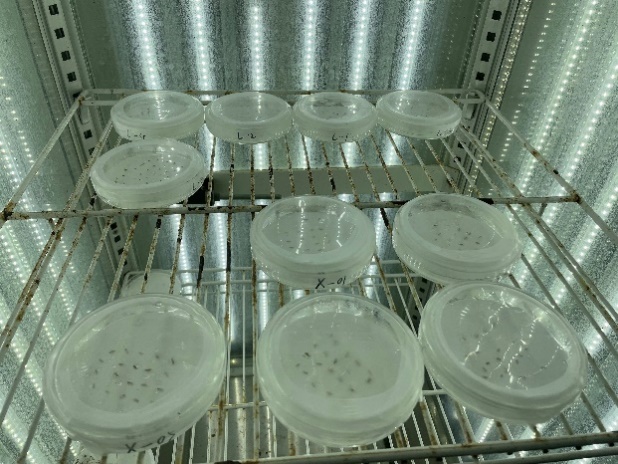

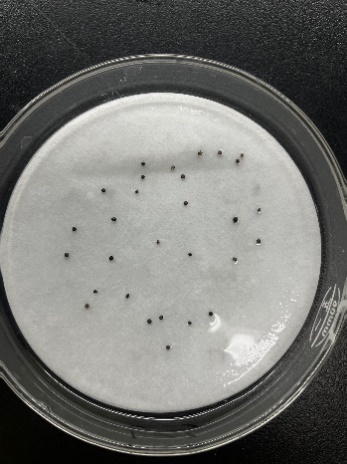
Figure S1 shows our pollination photos. We first selected 10 individuals with uniform growth, and 18 inflorescences were selected for each plant.

Figure S2: Photographs of germination experiments in an artificial incubator.

During the experiment, a 9cm petri dish with 2 layers of wet filter paper was used as the germination bed, and 25 seeds were uniformly placed in each dish. The experiment was carried out in an artificial climate chamber with a temperature of 25 ℃, relative humidity of 60%, 14 hours of light and 10 hours of darkness.
